# A New Alkamide with an Endoperoxide Structure from *Acmella ciliata* (Asteraceae) and Its *in Vitro* Antiplasmodial Activity

**DOI:** 10.3390/molecules21060765

**Published:** 2016-06-11

**Authors:** Narjara Silveira, Julia Saar, Alan Diego C. Santos, Andersson Barison, Louis P. Sandjo, Marcel Kaiser, Thomas J. Schmidt, Maique W. Biavatti

**Affiliations:** 1 Departamento de Ciências Farmacêuticas, Centro de Ciências da Saúde, Bloco J/K, Universidade Federal de Santa Catarina, Florianópolis 88040-900, SC, Brazil; silveira.narjara@gmail.com (N.S.); jsaar@hotmail.de (J.S.); p.l.sandjo@ufsc.br (L.P.S.); 2 Institut für Pharmazeutische Biologie und Phytochemie (IPBP), University of Münster, PharmaCampus, Corrensstraße 48, Münster D-48149, Germany; 3 Departamento de Química, Universidade Federal do Paraná, Curitiba 81530-900, PR, Brazil; alandiego@ufpr.br (A.D.C.S.); anderbarison@gmail.com (A.B.); 4 Swiss Tropical and Public Health Institute (Swiss TPH), Socinstr. 57, Basel CH-4051, Switzerland; marcel.kaiser@unibas.ch; 5 University of Basel, Petersplatz 1, Basel CH-4003, Switzerland

**Keywords:** *Acmella ciliata*, jambu, Asteraceae, antiplasmodial activity, alkamide, endoperoxide, *Plasmodium falciparum*

## Abstract

From the aerial parts of *Acmella ciliata* (H.B.K.) Cassini (basionym *Spilanthes ciliata* Kunth; Asteraceae), three alkamides were isolated and identified by mass- and NMR spectroscopic methods as (*2E*,*6E*,*8E*)-*N*-isobutyl-2,6,8-decatrienamide (spilanthol, (**1**)), *N*-(2-phenethyl)-2*E*-en-6,8-nonadiynamide (**2**) and (2*E*,7*Z*)-6,9-endoperoxy-*N*-isobutyl-2,7-decadienamide (**3**). While **1** and **2** are known alkamides, compound **3** has not been described until now. It was found that the unusual cyclic peroxide **3** exists as a racemate of both enantiomers of each alkamide; the 6,9-*cis*- as well as the 6,9-*trans*-configured diastereomers, the former represents the major, the latter the minor constituent of the mixture. *In vitro* tests for activity against the human pathogenic parasites *Trypanosoma brucei rhodesiense* and *Plasmodium falciparum* revealed that **1** and **3** possess activity against the NF54 strain of the latter (IC_50_ values of 4.5 and 5.1 µM, respectively) while **2** was almost inactive. Compound **3** was also tested against multiresistant *P. falciparum* K1 and was found to be even more active against this parasite strain (IC_50_ = 2.1 µM) with considerable selectivity (IC_50_ against L6 rat skeletal myoblasts = 168 µM).

## 1. Introduction

*Acmella ciliata* (H.B.K.) Cassini (basionym: *Spilanthes ciliata* Kunth; Asteraceae) is a herb native to tropical regions of South America [1]. Known under the common name jambu in Brazil, it is used as a spice and in traditional medicine for the treatment of toothaches and is the active ingredient in the herbal medicinal product Spolera^®^ used to treat abrasions and distortions [2]. Previous investigations have been carried out with this species that led to the isolation of several alkylamides (alkamides) [3,4], including the well-known bioactive isobutylamide spilanthol responsible for analgesic [5], anti-inflammatory [6] and antimalarial [7] *in vitro* activities.

The closely related species *Acmella oleracea* (L.) R. K. Jansen (basionym: *Spilanthes oleracea*; synonym: *Spilanthes acmella* var. *oleracea*) is widely used as spice for food and in folk medicine for stomatitis, cold and toothaches and is one of the plants constituting the traditional African antimalarial drug “Malarial-5” [8].

Malaria is a disease caused by various *Plasmodium* species and represents a public health problem causing many hundreds of thousands of deaths per year, especially to people living under poor circumstances and in vulnerable communities in tropical countries. According to the latest available data, an estimated 214 million cases occurred and the disease killed about 438,000 people in 2015 [9]. In this way, WHO recommends artemisinin-based combination therapies (ACTs) as the first line treatment of uncomplicated malaria caused by the *P. falciparum* parasite. However, since 2006, after the first artemisinin-resistant case was noticed in Cambodia, the resistance to artemisinin-derived drugs has become a global concern [10]. Therefore, the search for new antimalarial drugs has received increasing attention, including natural products. In a recent study, several alkamides from *Achillea ptarmica* (Asteraceae) were found to be active against *Plasmodium falciparum* and some other unicellular eukaryotic parasites such as *Trypanosoma brucei rhodesiense*, responsible for East African Human Trypanosomiasis [11]. Hence, an investigation of further alkamide-containing Asteraceae for alkamides with activity against such pathogens was of interest. Therefore, in the present investigation, we concentrated on alkamides of *Acmella ciliata* with potential activity against the mentioned parasites.

## 2. Results and Discussion

### 2.1. Isolation and Characterization of Alkamides

**Figure 1:**
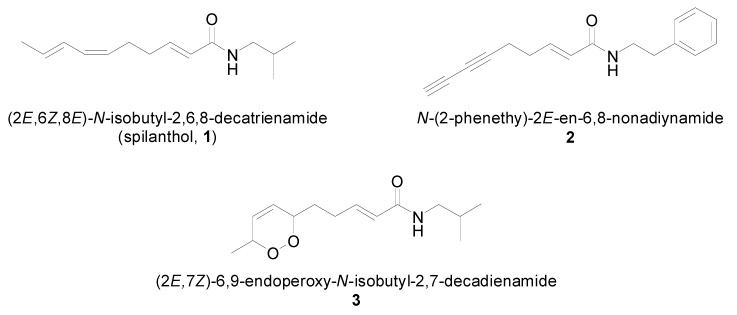
Structures of the isolated alkamides.

The hexane fraction of the ethanol extract from the fresh aerial parts of *Acmella ciliata* afforded, besides the well-known isobutylamide spilanthol ((*2E*,*6E*,*8E*)-*N*-isobutyl-2,6,8-decatrienamide, (**1**) and a further known compound *N*-(2-phenethyl)-2*E*-en-6,8-nonadiynamide (**2**), one new alkamide (2*E*,7*Z*)-6,9-endoperoxy-*N*-isobutyl-2,7-decadienamide (**3**) (Figure 1). Their chemical structures were fully established on the bases of 1D and 2D NMR and high-resolution mass spectrometric (HR-MS) data.

Compound **1** was obtained as a green oil and identified as spilanthol by comparison of its spectral data with those described in the literature [12].

Compound **2** was obtained as a yellow oil. It was previously isolated from *A. ciliata* [4] and its chemical structure was confirmed by comparison of the mass and ^1^H-NMR spectroscopic data with those reported in the literature.

**Figure 2:**
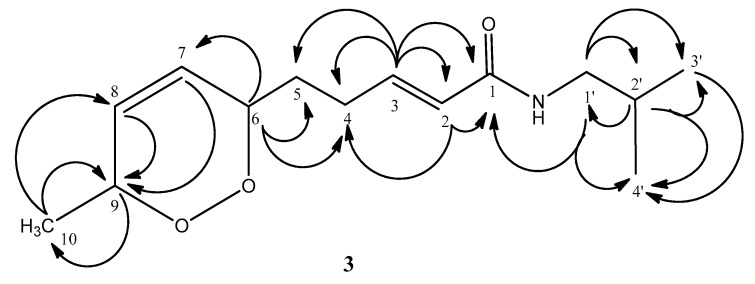
Key HMBC correlations for compound **3**.

Compound **3** was also obtained as a yellow oil. The +ESI QqTOF mass spectrum obtained under UHPLC coupling allowed establishment of the elemental formula as C_14_H_23_NO_3_ ([M + H]^+^ at *m*/*z* 254.1759, [M + Na]^+^ at *m*/*z* 276.1574; calcd. 254.1751 and 276.1570, respectively). While the isolate yielded one sharp peak in the UHPLC/MS chromatogram, it was found to show NMR signals of two very similar isomeric forms (ratio of approximately 3:1) in which most signals resonated at very similar or identical shift values with few exceptions. Nevertheless, the chemical shifts in the proton spectrum, even in parts of the spectrum with strong signal overlap, could all be determined with the help of a 2D J-resolved ^1^H-NMR experiment and all ^1^H/^1^H-coupling constants became accessible by selective homonuclear decoupling measurements. The NMR spectra (Table 1 and Table 2) showed the presence of an isobutylamide moiety with ^1^H signals at δ 3.14 (2H, H-1’), 1.80 (1H, H-2’) and 0.92 (6H, H-3’ and H-4’) and ^13^C resonances at δ 46.8 (C-1’), 28.5 (C-2’) and 20.1 (C-3’, C-4’) for the amine component as well as the amide carbonyl at 166.0 ppm (C-1), the latter correlated with the proton signal at δ 3.14 ppm in the HMBC spectrum (key HMBC correlations are summarized in Figure 2).

Two proton signals at δ 5.82 (1H, H-2) and 6.81 (1H, H-3) with a coupling constant of 15.2 Hz were attributed to an *E*-configured double bond. Their chemical shift values as well as the correlations observed in the HSQC and HMBC experiments showed that this double bond is in the direct neighborhood of the amide carbonyl, *i.e.*, that it is part of an α,β-unsaturated amide group such as that also observed in **1** and **2**. Further HMBC correlations of the two mentioned olefinic protons were observed with the methylene carbons at δ 27.9 (C-4) and 31.5 (C-5). The ^1^H resonances of two protons at an additional double bond were observed at δ 5.83 (H-7) and 5.86 (H-8), with a coupling constant of 10.3 Hz, indicating a *Z*-configuration. The corresponding olefinic carbon signals were observed at δ 127.0 and 129.3, respectively. Apart from the aforementioned signals, the ^1^H-NMR spectrum presented signals at δ 4.38 and 4.66 correlated according to the HSQC spectrum with carbon resonances at δ 77.2 (C-6) and 74.3 (C-9) in the ^13^C-NMR spectrum. These signals clearly show the presence of two oxygenated sp^3^ carbons in the structure. From the HMBC correlations it became clear that both of these carbons are direct neighbors of the *Z*-configured double bond, the former showing further correlations with the methylene signals of positions 4 and 5, the latter with a methyl group (position 10) resonating at δ 1.23 in the ^1^H- and at 18.1 ppm in the ^13^C-NMR spectrum.

The signals of the protons and carbons especially at this structural fragment (*i.e.*, positions 6–10) appeared to have shifted between the major and minor components (see below).

These NMR data were similar to those described for longipenamide B (*N*-isobutyl-*syn*-6,9-dihydroxy-2*E*,7*E*-decadienamide) known from *Heliopsis longipes* [14] which, however, has an elemental formula of C_23_H_25_NO_3_, *i.e.*, one degree of unsaturation less than the compound described here. Longipenamide B possesses an *E*-configured double bond between C-7 and C-8 and free hydroxy groups at C-6 and C-9. The lack of two hydrogen atoms in comparison with longipenamide B, together with the *Z*-configuration of the double bond indicated the presence of a cyclic structure in this part of the molecule which could then either be a dihydrofuran system, requiring the presence of one free hydroxy group, or a dihydro-1,2-dioxine system, *i.e.*, a cyclic peroxide structure. Since no HMBC correlations were observable either in chloroform-d_3_ or in acetone-d_6_ between the protons and carbons at positions 6 and 9, which would be expected in case of a furanoid system, the compound was indeed assigned the depicted structure of (2*E*,7*Z*)-6,9-endoperoxy-*N*-isobutyl-2,7-decadienamide. The observation that especially the NMR signals for the fragment between C-6 and C-10 were shifted between the major and minor isomers pointed towards the presence of two stereoisomers, *i.e.*, that one represents the *cis*-, the other the *trans*-configured form with respect to the asymmetric carbons, C-6 and C-9. It might be expected that the *cis*-oriented protons in the former would show an NOE correlation, as was previously described for a similar endoperoxide from *Zanthoxylum* [15]. However, NOEs could not be observed in the 2D NOESY spectrum for either isomer so that the relative stereochemistry could not be clarified in this manner. As mentioned above, the NMR signals of the protons around the endoperoxide ring showed significant shift differences in the minor isomer with H-6 being shifted downfield most prominently for Δδ 0.21 ppm, in comparison to the major isomer. Since no directly comparable data exist in literature, molecular modeling using quantum mechanical calculations had to be applied to assign the relative configuration for the major and minor isomers. To this end, the NMR shift values were calculated for geometry optimized models of the energetically most favorable conformers of both diastereomers (6*R*,9*R*-*trans* and 6*S*,9*R*-*cis*) of the de-alkylated carboxamide part of **3** (the isobutyl moiety was omitted to save computation time). The geometry optimized models were energy minimized with the B3LYP density functional and the 6-31++G(2d,p) basis set. The NMR shifts were calculated using the GIAO method at the same level of theory.

**Figure 3:**
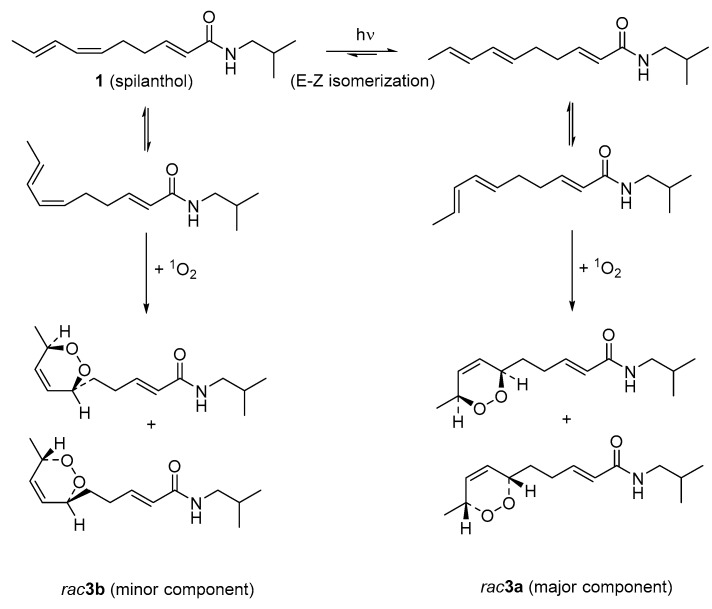
Hypothetical routes of formation for the stereoisomers of compound **3**.

**Figure 4:**
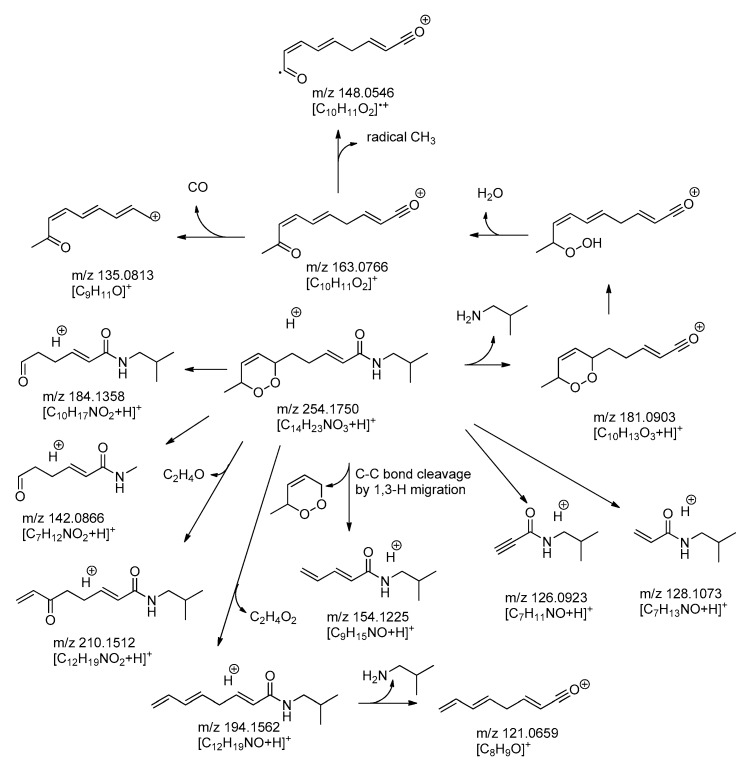
Proposed fragmentation mechanism of the endoperoxide alkamide (*m*/*z* 254).

As reported in Table 3, the shift differences resulting for the *cis*- and *trans*-diastereomers were in good qualitative agreement with the major (**3a**) and minor isomers (**3b**), respectively, of compound **3**. The formation of **3** (see Figure 3) is most likely to result from addition of a molecule of singlet oxygen to the diene moiety of the *E*,*E*-isomer of spilanthol (**1**) in case of the major isomer **3a**, and to **1** itself in case of the minor isomer **3b**. Light-induced isomerization of the 6,7-*Z*-8,9-*E* diene of **1** to the (energetically more favorable) *E*,*E*-isomer is in agreement with earlier literature reports on peroxidation of unsaturated fatty acids [15]. In order to evaluate whether compound **3** is a genuine product of the plant’s metabolism or formed during the extraction process, the fresh aerial parts (50 g) of *A. ciliata* were macerated with chloroform (100 mL) for 2 h in a flask protected from light with aluminum foil. The chloroform extract was dried *in* *vacuo* and its acetonitrile solution was then subjected to UPLC-ESI-MS analysis. Moreover, fresh flowers and leaves were quickly pounded and the resulting juices were probed by ASAP (Atmospheric solid analysis probe) MS performed according to established protocols as reported previously, e.g., in [16]. Both experiments displayed the pseudo-molecular ion *m*/*z* 254.1759 corresponding to **3** so that its formation is likely to occur already in the living plant to some extent. The structure was further supported by MS^2^ fragmentation as depicted in Figure 4. Taken together, it is suggested that addition of ^1^O_2_ proceeds in an uncatalyzed spontaneous manner. This reaction is possible by means of a 1,4-cycloaddition [17], so that the oxygen is added from either face of the molecules with equal probability (see Figure 3). Therefore, racemates should result in both cases. Indeed, the isolated mixture **3** was devoid of optical activity (no CD cotton effects between 450 and 190 nm) so that this result is in agreement with the postulated means of formation. It can thus be concluded that **3** is actually a mixture of all four possible stereoisomers with the 6*R*,9*S*- and 6*S*,9*R*-*cis* enantiomers present as major (**3a**, approximately 40% each) and the 6*R*,9*R*- and 6*S*,9*S*-enantiomers as the minor constituents (**3b**, approx. 10% each). Similar alkamides with an endoperoxide bridge have previously been described by Devkota *et al.* [15] as constituents of a *Zanthoxylum* species. However, the structure described here is a new natural product and has also not been described as a synthetic product, to the best of our knowledge. For this new compound, we propose the name dioxacmellamide.

### 2.2. In Vitro Antiplasmodial Activity of Isolated Alkamides

As part of our ongoing efforts to find new compounds with activity against “protozoan” parasites causing neglected tropical diseases as well as malaria, the isolated alkamides were tested for activity against *Trypanosoma brucei rhodesiense* (*Tbr*) and *Plasmodium falciparum* (*Pf*) as well as for cytotoxicity against mammalian cells (L6 cell line) using established protocols [18]. The resulting IC_50_ values for *in vitro* growth inhibition are reported in Table 4. While compound **2** displayed only insignificant activity against either parasite, compounds **1** and **3** were somewhat more active against *Tbr* and, especially *Pf* (chloroquine sensitive NF54 strain). In case of **1**, antiplasmodial activity at a comparable activity level against strains PFB and K1 of *Pf* had already been reported [7]. Compound **3**, due to its new endoperoxide structure that is related to the most important structure element of the potent antimalarial artemisinin, was also tested against the multi-resistant K1 strain of *Pf* where it displayed even higher activity than against the sensitive strain with an IC_50_ value of 0.54 µg/mL (2.1 µM) and showed considerable selectivity, *i.e.*, very low cytotoxicity against the L6 cells (S.I. = 78.7). Compound **3** thus appears to be an interesting candidate for *in vivo* tests against plasmodial infections which are warranted after isolation of larger quantities.

## 3. Materials and Methods

### 3.1. General Procedures

Silica gel 60 and Sephadex^®^ LH-20 (GE Healthcare, Uppsala, Sweden) were used for column chromatography, while silica gel 60 F_254_ aluminum sheets (Silicycle^®^, Ville de Québec, QC, Canada) were used for analytical thin layer chromatography (TLC). Compounds were visualized by exposure under UV_254/365_ light and by spraying with *p*-anisaldehyde reagent followed by heating. All solvents for chromatographic procedures were of analytical grade, while those for MS analysis were of spectrometric grade. NMR experiments were acquired at 303 K in CDCl_3_ and acetone-*d*_6_ on a Bruker (Billerica, MA, USA) AVANCE III NMR spectrometer and on an Agilent (Santa Clara, CA, USA) DD2 600 spectrometer, operating at 14.1 Tesla, observing ^1^H and ^13^C at 600 and 150 MHz, respectively. High-resolution MS and MS/MS analysis of the pure compounds were performed on a Bruker MicrOTOF-QII ESI-QTOF mass spectrometer coupled to a Dionex (Sunnyvale, CA, USA) Ultimate 3000 RS UHPLC. LC-MS and MS analyses of CHCl_3_ extract and the plant material were carried on a Waters (Milford, MA, USA) Xevo G2-S QTOF mass spectrometer equipped with exchangeable ESI and ASAP sources and coupled to an Acquity UPLC (Waters) chromatograph. A CD spectrum of **3**, recorded in MeOH on a Jasco (Groß-Umstadt, Germany) J815 spectropolarimeter, did not show any cotton effects.

### 3.2. Botanical Material

Aerial parts of *Acmella ciliata* (H.B.K.) Cassini were collected at the garden of the Associação dos Funcionários Fiscais do Estado de Santa Catarina (AFFESC, Florianópolis, Santa Catarina, Brazil) in October, 2012. The plant material was identified by Dr. Marcos Sobral and a voucher specimen was deposited in the Herbarium of the Botanic Garden of Rio de Janeiro under reference RB 612273.

### 3.3. Extraction and Isolation

**Figure 5:**
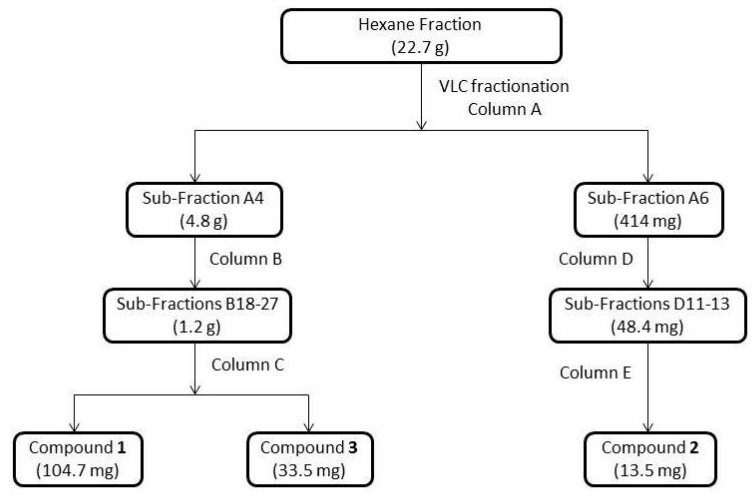
Flow chart for the isolation of the compounds **1**–**3**.

A summary of the isolation procedure for compounds **1**–**3** is shown in Figure 5. Aerial parts of fresh plant (1754 g) were washed with water, air dried and macerated for 3 × 7 days with ethanol 92% (total volume 8 L) at room temperature. The crude extract was filtered and concentrated under reduced pressure at 40 °C, yielding 60.9 g of crude ethanol extract. The extract was then dispersed in water and partitioned with solvents of increasing polarity: *n*-hexane (24.4 g), followed by CH_2_Cl_2_ (0.8 g), and EtOAc (1.6 g), and the residual aqueous fraction was freeze-dried.

The hexane fraction (22.7 g) was initially fractionated by VLC packed with silica gel 60 (particle size 40–63 µm) (Column A) and eluted with gradient solvent systems of *n*-hexane/acetone in increasing polarities (100:0 (350 mL), 90:10 (300 mL), 80:20 (400 mL), 70:30 (400 mL), 60:40 (600 mL), 40:60 (300 mL), 20:80 (200 mL), 0:100 (300 mL), respectively), yielding 13 sub-fractions. Sub-fraction A4 (4.8 g) was subjected to a silica gel 60 (particle size 40–63 µm) column chromatography (Column B) and eluted with gradient solvent systems of *n*-hexane/acetone in increasing polarity (100:0 (200 mL), 98:02 (100 mL), 95:05 (500 mL), 92:08 (300 mL), 90:10 (100 mL), 85:15 (400 mL), 80:20 (100 mL), 70:30 (200 mL), 60:40 (100 mL), 0:100 (100 mL), respectively), yielding 48 sub-fractions. The sub-fractions B18–B27 (1.2 g) were submitted to another silica gel 60 (particle size 40–63 µm) column chromatography (Column C) and eluted with gradient solvent systems of *n*-hexane/acetone in increasing polarity (100:0 (50 mL), 95:05 (100 mL), 90:10 (50 mL), 80:20 (50 mL), 75:25 (100 mL), 60:40 (100 mL), 50:50 (50 mL), 40:60 (100 mL), 0:100 (50 mL), respectively), yielding 18 sub-fractions. Compound **1** was obtained from sub-fractions C7–C8 (104.7 mg, green oil) and compound **3** was obtained from sub-fractions C11–C12 (35.5 mg, yellow oil). Sub-fraction A6 (414 mg) from Column A was subjected to a silica gel 60 (40–63 µm) column chromatography (Column D) and eluted with gradient solvent systems of *n*-hexane–acetone in increasing polarity (80:20 (500 mL), 70:30 (200 mL), 0:100 (50 mL), respectively), yielding 25 sub-fractions. The sub-fractions D11–D13 (48.4 mg) were submitted to a Sephadex LH20 column chromatography (Column E) and eluted with methanol yielding 16 sub-fractions. Compound **2** was obtained from subfraction E7 (13.5 mg, yellow oil). All sub-fractions were monitored by analytical TLC on silica gel 60 F_254_ aluminum sheets using *n*-hexane:acetone (6:4; *v*/*v*) as mobile phase; detection was performed at daylight, 366 and 254 nm, and after spraying with *p*-anisaldehyde reagent followed by heating. The fractionation and isolation process is summarized in Figure 4.

### 3.4. Biological Activity Tests

*In vitro* tests for activity against *T. brucei rhodesiense* (bloodstream trypomastigotes, strain STIB 900) and *P. falciparum* (intraerythrosctic forms, strains NF54 and K1) as well as for cytotoxicity against mammalian cells (L6 rat skeletal myoblasts) were performed according to established protocols as reported previously, e.g., in [18].

### 3.5. Molecular Modeling and NMR Shift Calculations for Compound ***3***

All calculations were performed with molecular models of the dealkylated carboxamide part of **3**, *i.e.*, the isobutyl residue was omitted in order to save computation time. A molecular model of each, the 6*S*,9*R*-*cis*- and the 6*R*,9*R*-*trans*- diastereomer was generated with the software package MOE [19] and submitted to a low-mode molecular dynamics conformational search using the MMFF94x force field and standard settings. The energetically lowest conformer of each stereoisomer found in these searches was energy-minimized with Gaussian 03W [20] using the B3LYP density functional and the 6-31G++(2d,p) basis set. Subsequently, the NMR shielding tensors were computed with the same software and at the same level of theory using the GIAO formalism. A molecule of tetramethylsilane was treated in the same manner in order to obtain the reference value (34.9441 ppm). Chemical shift values were obtained by subtracting the TMS reference value from the values calculated for the protons mentioned in Table 1.

## 4. Conclusions

The search for alkamides with anti-protozoal activity in the medicinal plant *Acmella ciliata* has resulted in the discovery of dioxacmellamide (**3**), a new alkamide with cyclic peroxide structure which shows considerable *in vitro* activity against *P. falciparum* with selectivity against the multiresistant K1 strain. It is very likely that this interesting compound is formed by 1,4-cycloaddition of oxygen to the diene moiety of the plant product spilanthol, and it could also be demonstrated that it can easily be obtained from the latter. This accessibility from the main constituent spilanthol will enable us to obtain larger quantities for *in vivo* tests and detailed studies of the mechanism of action of this new anti-plasmodial hit molecule. Such studies have been initiated.

## Figures and Tables

**Table 1:** ^1^H-NMR data (CDCl_3_, 600 MHz) for compounds **3a** and **3b**.

Position	3a	3b
	δ_H_, Mult. (*J* in Hz)	δ_H_, Mult. (*J* in Hz)
**1**		
**2**	5.82 *ddd* (15.2, 1.4 and 1.4)	5.82 *ddd* (15.2, 1.4 and 1.4)
**3**	6.81 *ddd* (15.2, 7.4 and 6.6)	6.81 *ddd* (15.2, 7.4 and 6.6)
**4**	2.32 *ddddd* (14.9, 8.8, 7.4, 7.2 and 1.4)	2.32 *ddddd* (14.9, 8.8, 7.4, 7.2 and 1.4)
	2.36 *ddddd* (14.9, 9.1, 6.6, 5.3 and 1.4)	2.36 *ddddd* (14.9, 9.1, 6.6, 5.3 and 1.4)
**5**	1.69 *dddd* (14.2, 9.1, 7.2 and 4.0)	1.67 *dddd* (14.2, 9.1, 7.2 and 4.0)
	1.87 *dddd* (14.2, 8.9, 8.8 and 5.3)	1.87 *dddd* (14.2, 8.9, 8.8 and 5.3)
**6**	4.38 *ddddd* (8.9, 4.0, 2.4, 1.3 and 1.2)	4.59 *ddddd* (8.9, 4.0, 2.4, 1.3 and 1.2)
**7**	5.83 *ddd* (10.3, 2.4 and 1.5)	5.78 *ddd* (10.3, 2.4 and 1.5)
**8**	5.86 *ddd* (10.3, 1.7 and 1.2)	5.83 *ddd* (10.3, 1.7 and 1.2)
**9**	4.66 *qddd* (6.7, 1.7, 1.5 and 1.3)	4.70 *qddd* (6.7, 1.7, 1.5 and 1.3)
**10**	1.23 *d* (6.7)	1.20 *d* (6.7)
**1’**	3.14 *dd* (6.8 and 6.2)	3.14 *dd* (6.8 and 6.2)
**2’**	1.80 *th* (6.8 and 6.7)	1.80 *th* (6.8 and 6.7)
**3’**	0.92 *d* (6.7)	0.92 *d* (6.7)
**4’**	0.92 *d* (6.7)	0.92 *d* (6.7)

The corrected multiplicities in the ^1^H-NMR spectra were established with the aid of FOMSC3 software [13].

**Table 2:** ^13^C-NMR data (CDCl_3_, 150 MHz) for compounds **3a** and **3b**.

Position	3a		3b	
	δ_C_	HMBC	δ_C_	HMBC
**1**	166.0		165.9	
**2**	124.4	C-1, C-4	124.4	C-1, C-4
**3**	143.3	C-1, C-2, C-4, C-5	143.2	C-1, C-2, C-4, C-5
**4**	27.9	C-2, C-3, C-5, C-6	27.5	C-2, C-3, C-5, C-6
		C-2, C-3, C-5, C-6		C-2, C-3, C-5, C-6
**5**	31.5	C-3, C-4, C-6, C-7	31.0	C-3, C-4, C-6, C-7
		C-3, C-4, C-6, C-7		C-3, C-4, C-6, C-7
**6**	77.2	C-4, C-5, C-7, C-8	76.8	C-4, C-5, C-7, C-8
**7**	127.0	C-6, C-9	127.2	C-6, C-9
**8**	129.3	C-6, C-9	129.5	C-6, C-9
**9**	74.3	C-7, C-8, C-10	74.2	C-7, C-8, C-10
**10**	18.1	C-8, C-9	17.7	C-8, C-9
**1’**	46.8	C-1, C-2’, C-3’, C-4’	46.7	C-1, C-2’, C-3’, C-4’
**2’**	28.5	C-1’, C-3’, C-4’	28.5	C-1’, C-3’, C-4’
**3’**	20.1	C-1’, C-2’, C-4’	20.1	C-1’, C-2’, C-4’
**4’**	20.1	C-1’, C-2’, C-3’	20.1	C-1’, C-2’, C-3’

**Table 3:** Calculated and experimental chemical shift values and differences for protons at the endoperoxide ring of compound **3**. Upfield shifts comparing the first and second columns of calculated and experimental data, respectively, are colored in blue, downfield shifts in red.

Position H-	Calc. Δ		Δδ Calc	Exp. Δ		Δδ Exp
	6*S*,9*R*-*cis*	6*R*,9*R*-*trans*		Major Isomer	Minor Isomer	
5a	1.426	1.486	0.059	1.873	1.873	0.000
5b	2.149	1.510	−0.638	1.693	1.669	−0.024
6	4.030	4.622	0.591	4.377	4.586	0.209
7	6.098	5.859	−0.240	5.830	5.782	−0.048
8	6.032	5.950	−0.082	5.860	5.830	−0.030
9	4.807	4.849	0.042	4.663	4.696	0.033
10	0.982	0.998	0.016	1.232	1.203	−0.029

**Table 4:** *In vitro* antiparasitic activity IC_50_ values in µg/mL and cytotoxicity (L6 rat skeletal myoblasts) of alkamides **1**–**3**. Mean values are also reported in µM (in brackets) for easier comparison between compounds.

Compound	*Tbr*	*Pf* (NF54)	*Pf* (K1)	L6
**1**	2.88 ± 0.06 (13.0)	0.99 ± 0.12 (4.5)	n.t.	39.9 ± 1.9 (180.3)
**2**	17.1 ± 2.3 (68.1)	22.1 ± 4.0 (88.0)	n.t.	60.1 ± 9.4 (239.2)
**3**	5.60 ± 0.67 (13.0)	1.29 ± 0.20 (5.1)	0.54 ± 0.14 (2.1)	42.6 ± 1.7 (168.2)
Pos. contr.	0.004 ± 0.001 ^a^	0.002 ± 0.001 ^b^	0.09 ± 0.006 ^b^	0.004 ± 0.001 ^c^

^a^ melarsoprol; ^b^ chloroquine; ^c^ podophyllotoxin.

## Supplementary Materials

MS and NMR spectra of **3** as well as details on the MS analyses are available online at: http://www.mdpi.com/1420-3049/21/6/765/s1.
