# Supplementary material for: A New Alkamide with an Endoperoxide Structure from Acmella ciliata (Asteraceae) and Its in Vitro Antiplasmodial Activity
Source: Molecules. 2016 Jun 11;21(6):765. doi: 10.3390/molecules21060765 (PMC6274185; doi:10.3390/molecules21060765)
Supplement: Supplementary file 1 [file molecules-21-00765-s001.pdf]

Supplementary Materials: A New Alkamide with An Endoperoxide Structure from *Acmella ciliata* (Asteraceae) and Its *in Vitro* Antiplasmodial Activity

Narjara Silveira, Julia Saar, Alan Diego C. Santos, Andersson Barison, Louis P. Sandjo, Reto Brun, Marcel Kaiser, Thomas J. Schmidt and Maique W. Biavatti

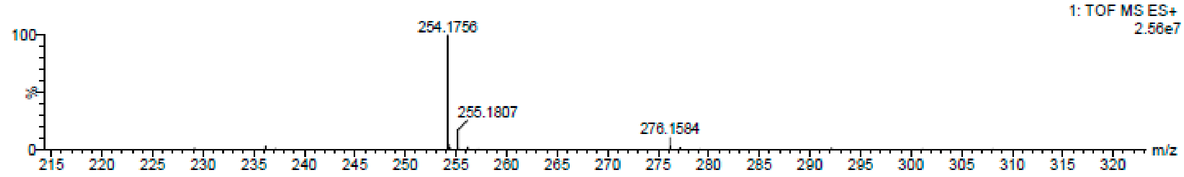

Mass	Calc. Mass	mDa	PPM	DBE	Formula
254.1756	254.1756	0.0	0.0	3.5	C14 H24 N O3

Figure S1. +ESI-QTOF mass spectrum of compound **3**.

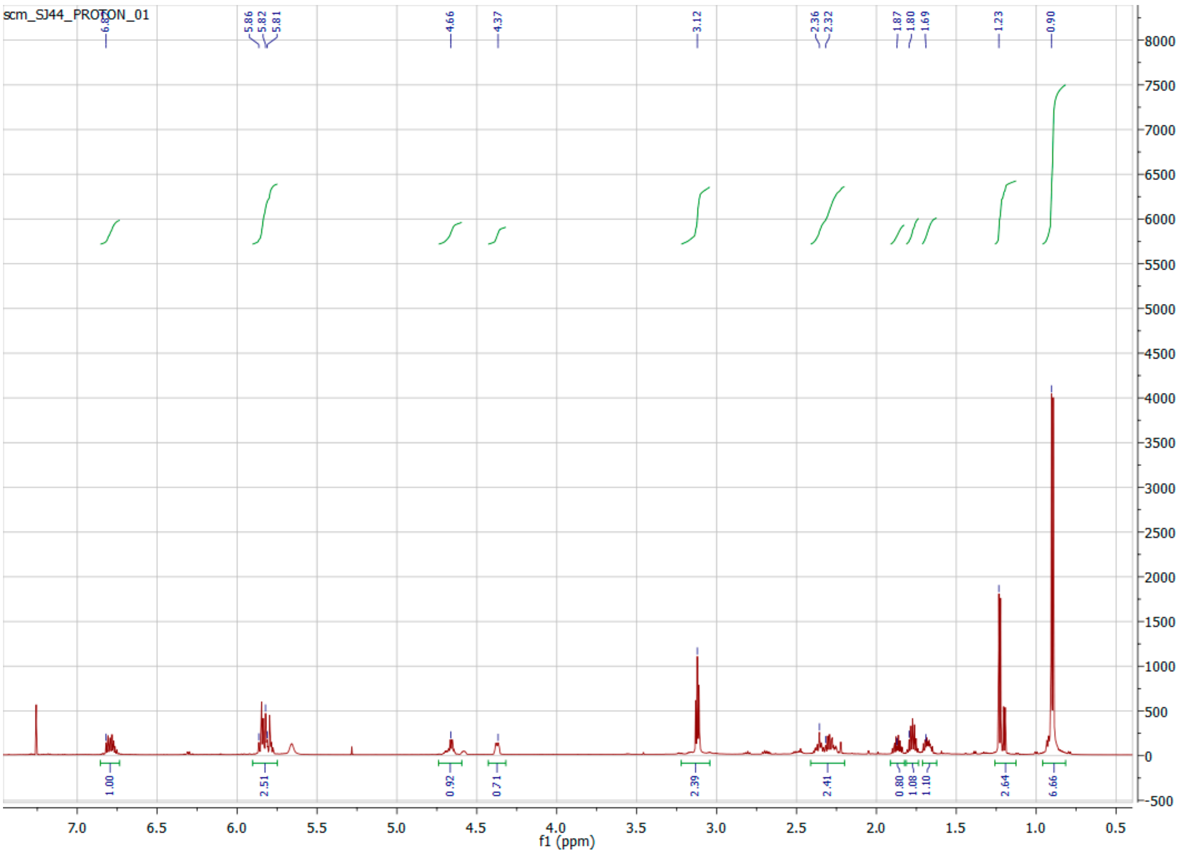

Figure S2. ^{1}H-NMR spectrum (600 MHz, $CDCl_3$) of compound **3**.

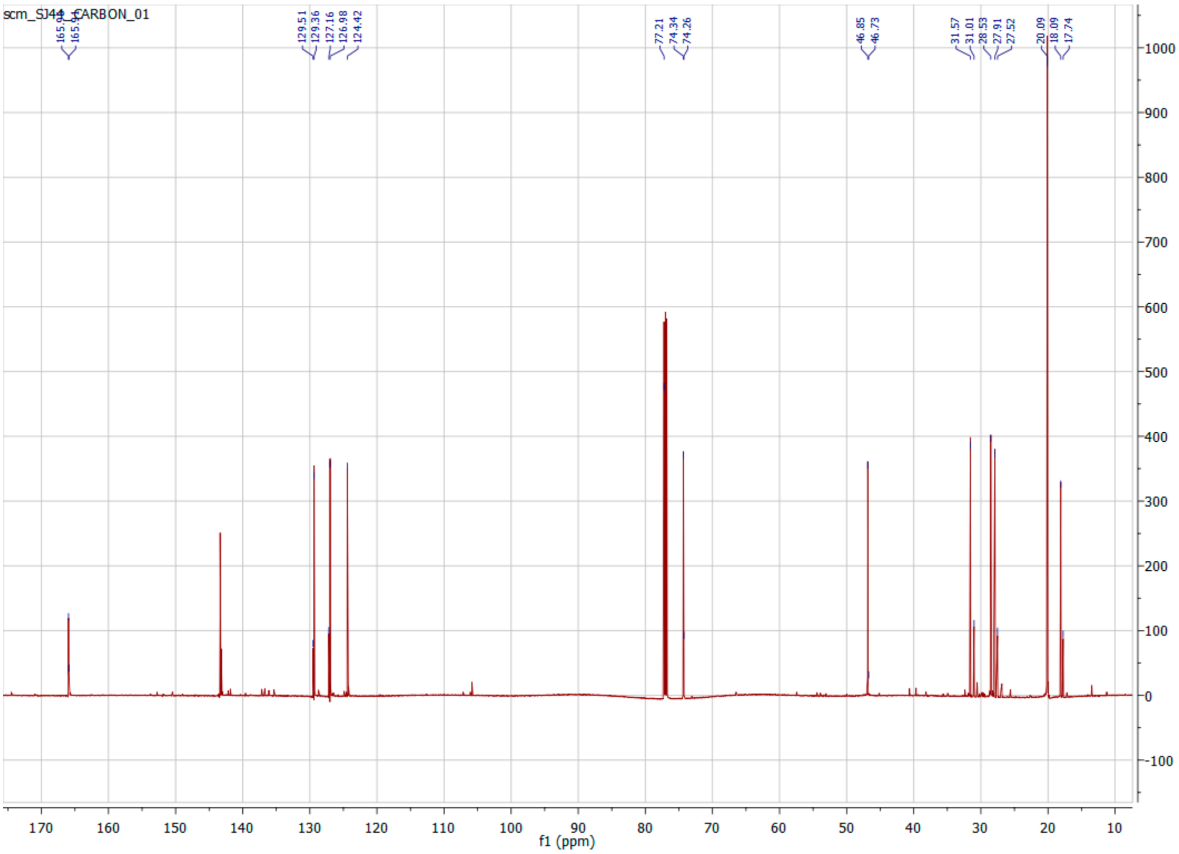

Figure S3. ^{13}C-NMR spectrum (150 MHz, $CDCl_3$) of compound **3**.

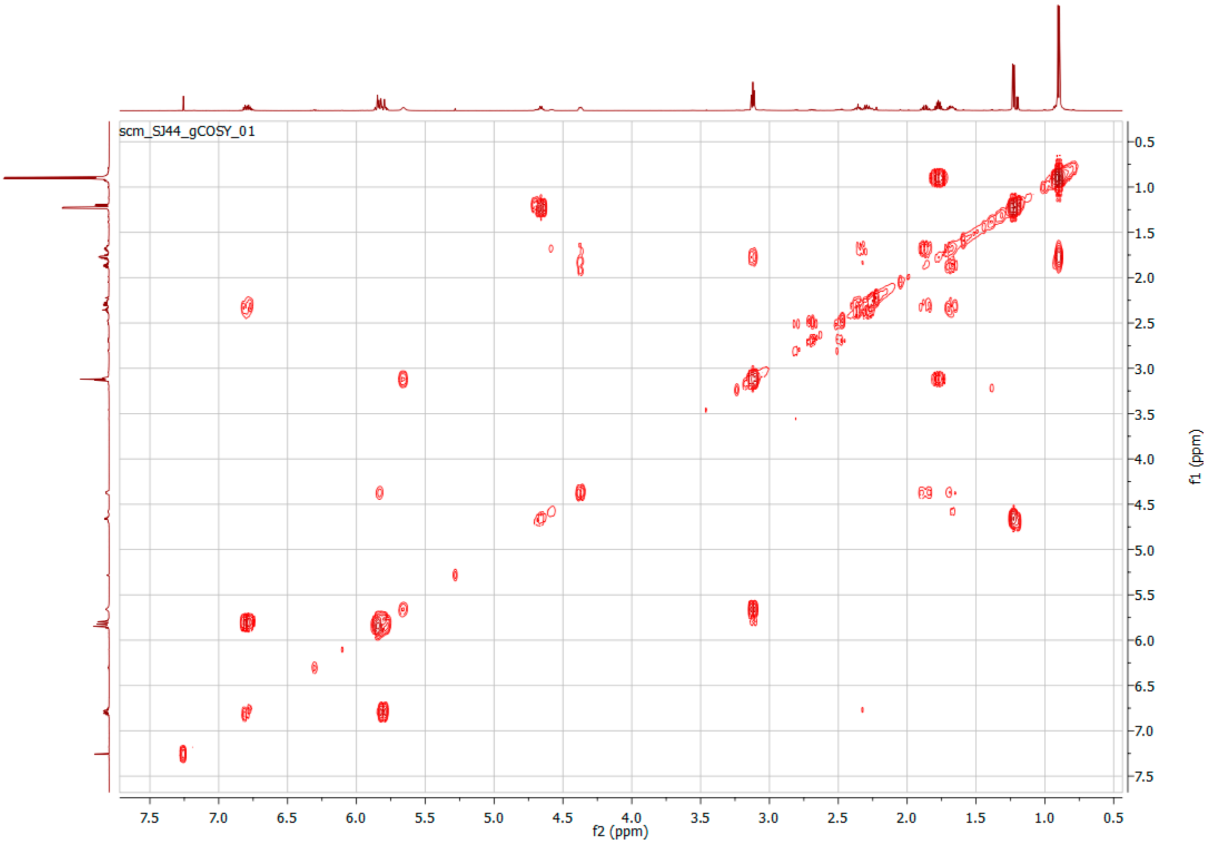

Figure S4. ^{1}H/^{1}H-COSY spectrum (600 MHz, $CDCl_3$) of compound **3**.

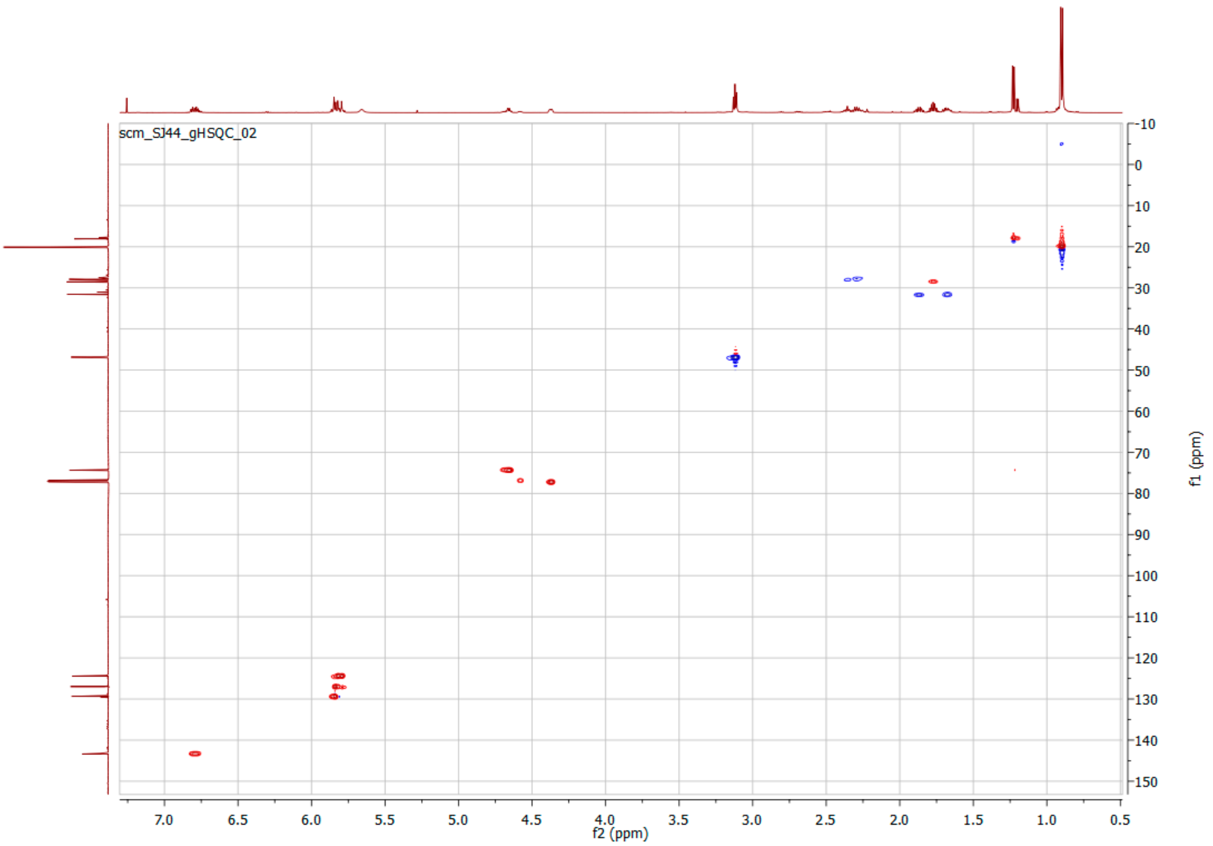

Figure S5. $^1H/^{13}C$-HSQC spectrum (600/150 MHz, $CDCl_3$) of compound **3**.

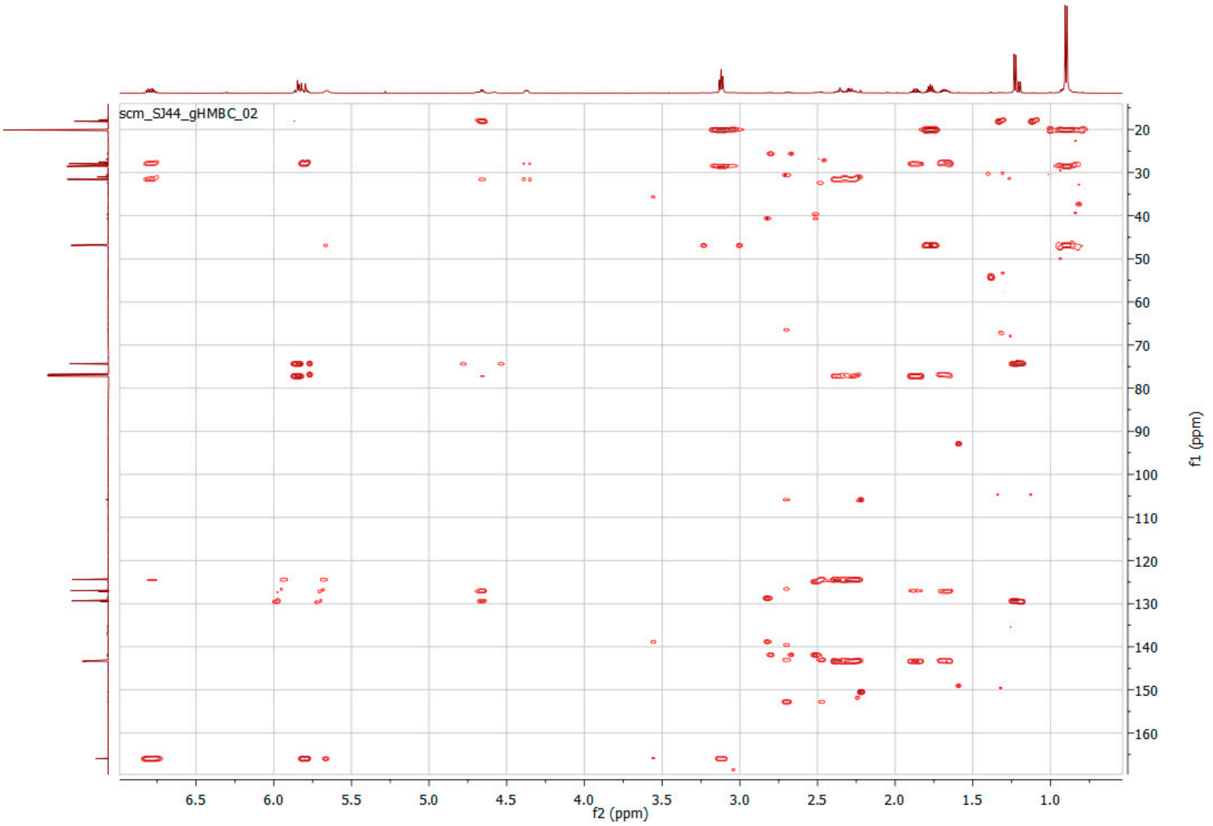

Figure S6. $^1H/^{13}C$-HMBC spectrum (600/150 MHz, $CDCl_3$) of compound **3**.

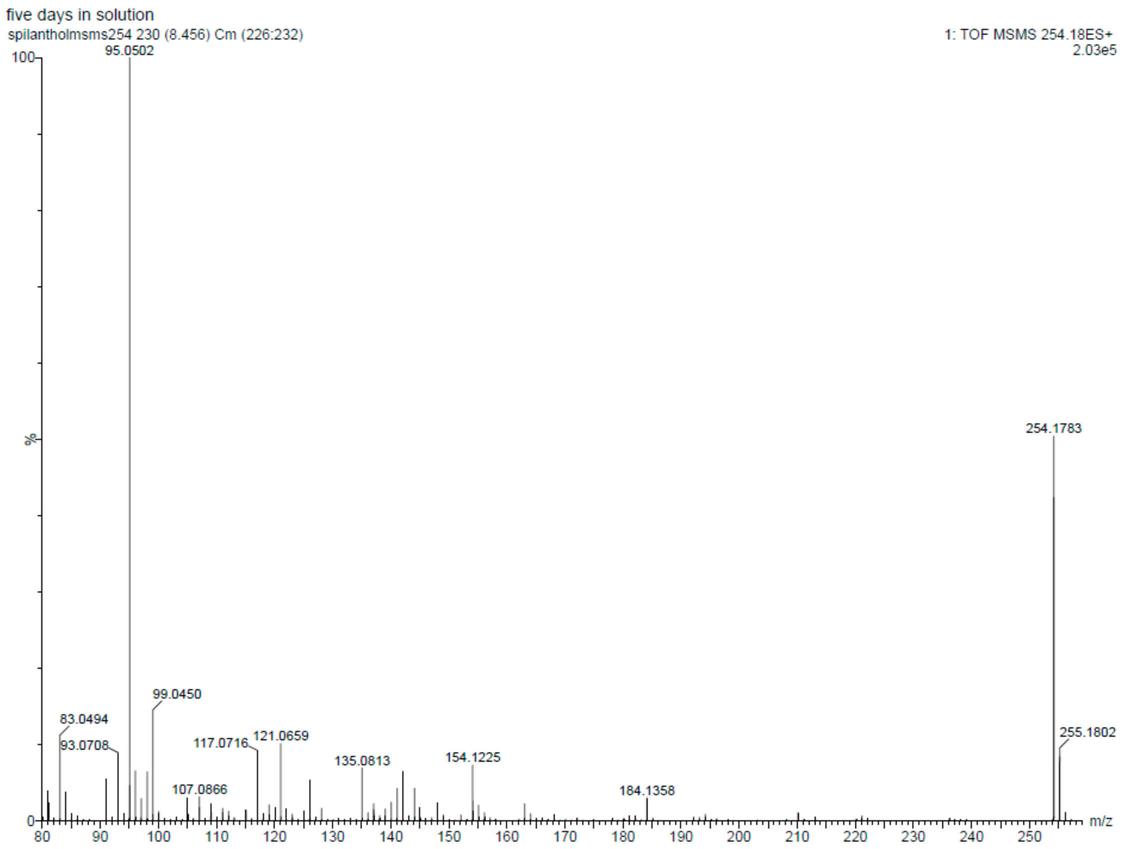

Figure S7. HR-ESI-MS/MS spectrum of the endoperoxide alkamide **3** (parent ion *m*/*z* 254).

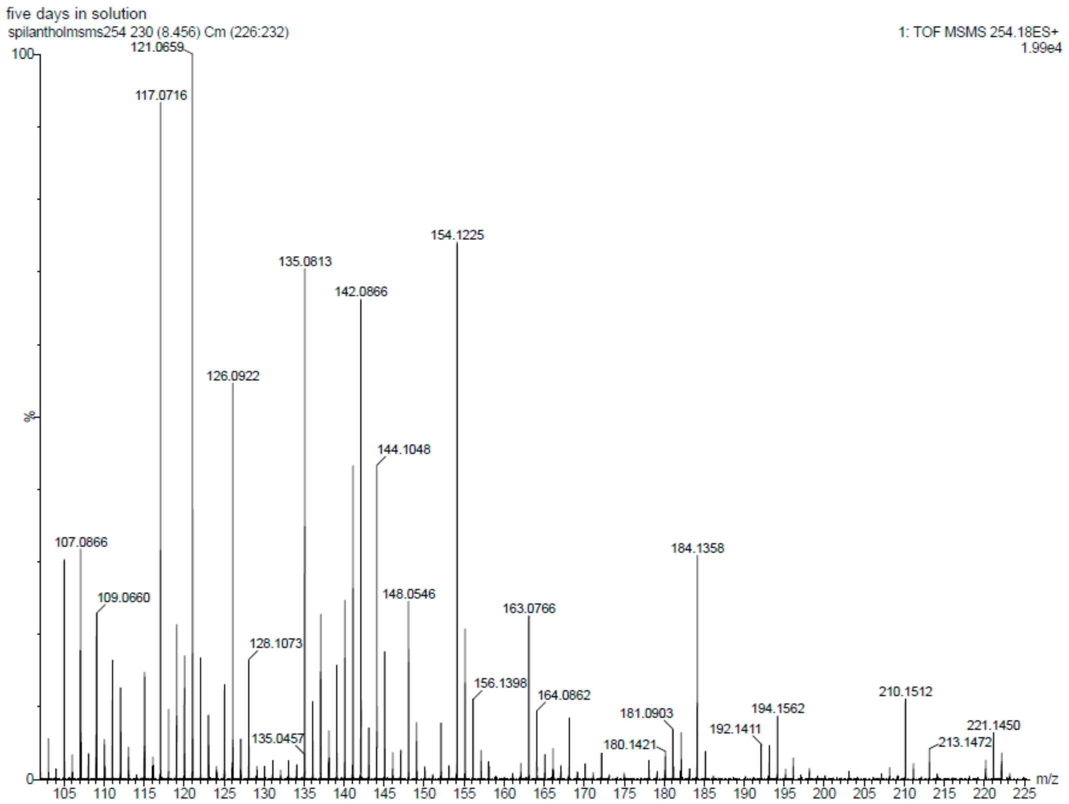

Figure S8. Magnified HR-ESI-MS/MS spectrum of the endoperoxide alkamide **3** (parent ion *m*/*z* 254).

LC-MS and LC-MS/MS Analyses

Pure compounds: Chromatograpic separation and check for purity was performed on a Dionex Ultimate 3000 RS Liquid Chromatography System on a Dionex Acclaim RSLC 120, C18 column (2.1 × 100 mm, 2.2 μm) with a binary gradient (A: water with 0.1% formic acid; B: acetonitrile with 0.1% formic acid) at 0.8 mL/min: 0 to 9.5 min: linear from 5% B to 100% B; 9.5 to 12.5 min: isocratic 100% B; 12.5 to 12.6 min: linear from 100% B to 5% B; 12.6 to 15 min: isocratic 5% B. The injection volume was 2 μL. Eluted compounds were detected using a Dionex Ultimate DAD-3000 RS over a wavelength range of 200–400 nm and a Bruker Daltonics micrOTOF-QII time-of-flight mass spectrometer equipped with an Apollo electrospray ionization source in positive mode at 5 Hz over a mass range of *m*/*z* 50–1,000 using the following instrument settings: nebulizer gas nitrogen, 5 bar; dry gas nitrogen, 9 L/min, 220 °C; capillary voltage 4,500 V; end plate offset −500 V; transfer time 70 μs; collision gas nitrogen; collision energy and collision RF settings were combined to each single spectrum of 1000 summations as follows: 250 summations with 20% base collision energy and 130 Vpp + 250 summations with 100% base collision energy and 500 Vpp + 250 summations with 20% base collision energy and 130 Vpp + 250 summations with 100% base collision energy and 500 Vpp. Base collision energy was 50 eV for precursor ions with a m/z less than 500 and then linearly interpolated against m/z up to a maximum of 70 eV for precursor ions with a *m*/*z* of up to 1000. Internal dataset calibration (HPC mode) was performed for each analysis using the mass spectrum of a 10 mM solution of sodium formiate in 50% isopropanol that was infused during LC re-equilibration using a divert valve equipped with a 20 μL sample loop. The sample concentration was 0.1 mg/mL in methanol.

CH_3Cl_3-extract: Chromatographic separation was performed on a BEH C18 column: 50 mm, 1.0 mm, particle size 1.7 μm (Waters) during 15 min. The temperature of the column and the sample tray temperature were respectively 40 °C and 20 °C. The gradient employed at flow rate of 0.3 mL/min, was 95% A (water/formic acid, 99.9/0.1 *v*/*v*) and 5% B (acetonitrile); 0–8 min, 60% of A; 8.1–8.5 min 40% of A; 8.5–10.9 min 10% of A; 10.9–15 min 95% of A. The injection volume of the sample was amounted to 5 μL. The analysis was recorded in ESI positive mode using the following instrument settings: nebulizer and desolvation gas: nitrogen; flow cone gas 10 L/h; flow desolvation gas 700 L/h; sampling cone 40 V; source offset 80 V; temperature cone 90 °C; temperature desolvation 200 °C; collision gas: argon; energy of collision: 25 eV; Lockspray reference sample: Leucine encephalin with the lock mass at *m*/*z* 556.2771 (ESI+).
